# Periodontal disease: Repercussions in pregnant woman and newborn health—A cohort study

**DOI:** 10.1371/journal.pone.0225036

**Published:** 2019-11-22

**Authors:** Marina Guim Otsuka Padovan Figueiredo, Stefanie Yaemi Takita, Bianca Maria Ramos Dourado, Helderjan de Souza Mendes, Erick Olsen Terakado, Hélio Rubens de Carvalho Nunes, Cátia Regina Branco da Fonseca

**Affiliations:** 1 Pediatrics Department, Botucatu Medical School, São Paulo State University (UNESP),Botucatu city, São Paulo State, Brazil; 2 Botucatu Clinics Hospital, Botucatu city, São Paulo State, Brazil; 3 Psychology Department, Paulista University (UNIP), São Paulo city, São Paulo State, Brazil; Stony Brook University Health Sciences Center School of Medicine, UNITED STATES

## Abstract

The periodontal disease during pregnancy triggers an exacerbated immune response with high local and systemic concentrations of inflammatory markers.

**Objective:**

To investigate the repercussion of periodontal disease (PD) in the pregnant woman health and the complications during pregnancy and delivery, as well as negative outcomes for the newborn (as infections, prematurity, low birth weight and fetal growth restriction).

**Method:**

Retrospective cohort study, based on medical records of 142 pregnant women assisted at a prenatal service of usual risk between 2012–2014, with a dental evaluation for PD. Maternal variables, along with labor and newborn variables, were analyzed. The newborns were stratified into two groups: offspring of mothers with PD (subdivided into Severe Periodontal Disease—SPD) and offspring of mothers without PD. Each outcome was adjusted by a multiple logistic regression model, with significance for p-value <0.05, considering all potential confounding factors.

**Results:**

Among women diagnosed with SPD, the odds ratio for vulvovaginitis was 3.45 times greater (OR = 3.45, p-value = 0.050) and 5.59 times higher for premature rupture of membranes (OR = 5.59; p-value = 0.017). For neonates, the chance of fetal growth restriction was 11.53 times higher for pregnant women with SPD (OR = 11.53, p = 0.041).

**Conclusion:**

The periodontal disease increased the chance of neonatal and maternal negative outcomes, being the fetal growth restriction, vulvovaginitis and premature rupture of the membrane (PROM) the main results driven by the presence of Severe Periodontal Disease.

## Introduction

The periodontal disease (PD) is a chronic infection caused by a bacteria that stimulates the immune-inflammatory response, leading to inflammation of the gingival and supportive tissue of the teeth, resulting from the pathogenesis of the microorganism and the response of the host. The main manifestations of the disease are gingivitis with an initial inflammatory response and periodontitis—or severe periodontal disease (SPD) -, resulting from the long exposure to the inflammation and identified by the formation of calculi and periodontal pockets that may lead to the destruction of the periodontal ligament [1].

The systemic response to PD is due to the release of metalloproteinase and prostaglandins, mainly PgE2 [2], which stimulates the production of cytokines and pharmacologically active mediators, such as interleukin 1-beta, IL-6, alpha tumoral necrosis factor, there is the action of osteoclasts and dental bone resorption. These cytokines and infectious agents can spread systemically through the bloodstream [3].

In pregnant women, high concentrations of estrogen and progesterone predispose the PD [4]; when associated with oral hygiene deficiency of a population, PD can affect up to 60% of this group [5]. PD during pregnancy may trigger an exacerbated immune response with high local and systemic concentrations of inflammatory markers [6] and thereby increase the risk of systemic alterations.

Studies have investigated an association between periodontal conditions and possible complications for the pregnant woman and the newborn, such as Offenbacher *et al*. (1998), which indicated the association between this disease and preterm delivery [3]. Several complications associated with PD, such as gestational diabetes, preeclampsia, intrauterine growth restriction, early abortion, preterm birth, low birth weight and a higher risk of early neonatal infection are reported in the literature. However, there is still no agreement on the actual effects of PD in this group.

Considering this scenario, investigating the repercussion of PD for the pregnant woman, as well as negative outcomes for the newborn, is of utmost importance for the health care.

Our primary objective in this study is to investigate possible repercussions of the periodontal disease during pregnancy and labor (preterm labor, gestational hypertensive disease, preeclampsia and eclampsia), as well as alterations involving the newborns. We also intend to determine if the severity of the periodontal disease influences these repercussions.

## Materials and methods

### Study design

Retrospective cohort study with pregnant women assisted at a prenatal service of usual risk, previously evaluated by a dentist at the Basic Health Unit (BHU) for the presence of PD, from 2012 to 2014, in the city of Botucatu, State of São Paulo, Brazil [7].

The study includes pregnant women from the 16th week until the end of gestation, all in good general health conditions and without any habitual gestational risks. The analysis is based on a random sampling of pregnant women assisted in every BHU, considering the importance of each BHU in relation to the total number of pregnant women attended in 2011 (Table 1). All participants in this study had to give consent for themselves following Brazilian Medical Research Council guidance [8].

**Table 1:** Number and percentage of the 142 pregnant women included in the study according to their distribution in each health unit of the city of Botucatu—SP.

Health Unit	N° pregnant women -2011	Statistical weight	Number	Percentage (%)
1.	172	0.106	16	11.3
2.	139	0.085	12	8.5
3.	160	0.098	12	8.5
4.	145	0.089	12	8.5
5.	128	0.078	11	7.7
6.	157	0.096	13	9.2
7.	101	0.062	09	6.3
8.	109	0.067	08	6.3
9.	111	0.068	10	7.0
10.	87	0.053	08	5.6
11.	83	0.051	07	4.9
12.	78	0.047	07	4.9
13.	84	0.052	08	5.7
14.	39	0.030	05	3.5
15.	28	0.016	03	2.1

Source: Oral health conditions of pregnant woman in Botucatu / São Paulo state, Brazil [7].

All the pregnant women who gave birth at the Botucatu Clinics Hospital (HCFMB) were included in the study and were considered "losses" only those whose personal data could not be retrieved—after exhaustive attempts to check the electronic medical records of the Hospital, the physical records at the Botucatu Basic Health Units (BHU) and the Declaration of Live Births. It is worth noting that the Maternity of HCFMB is the only public maternity of the municipality. The Table 1 shows the number and percentage of wich BHU, and the name of each one was changed by number so as not identify them.

**Fig 1:**
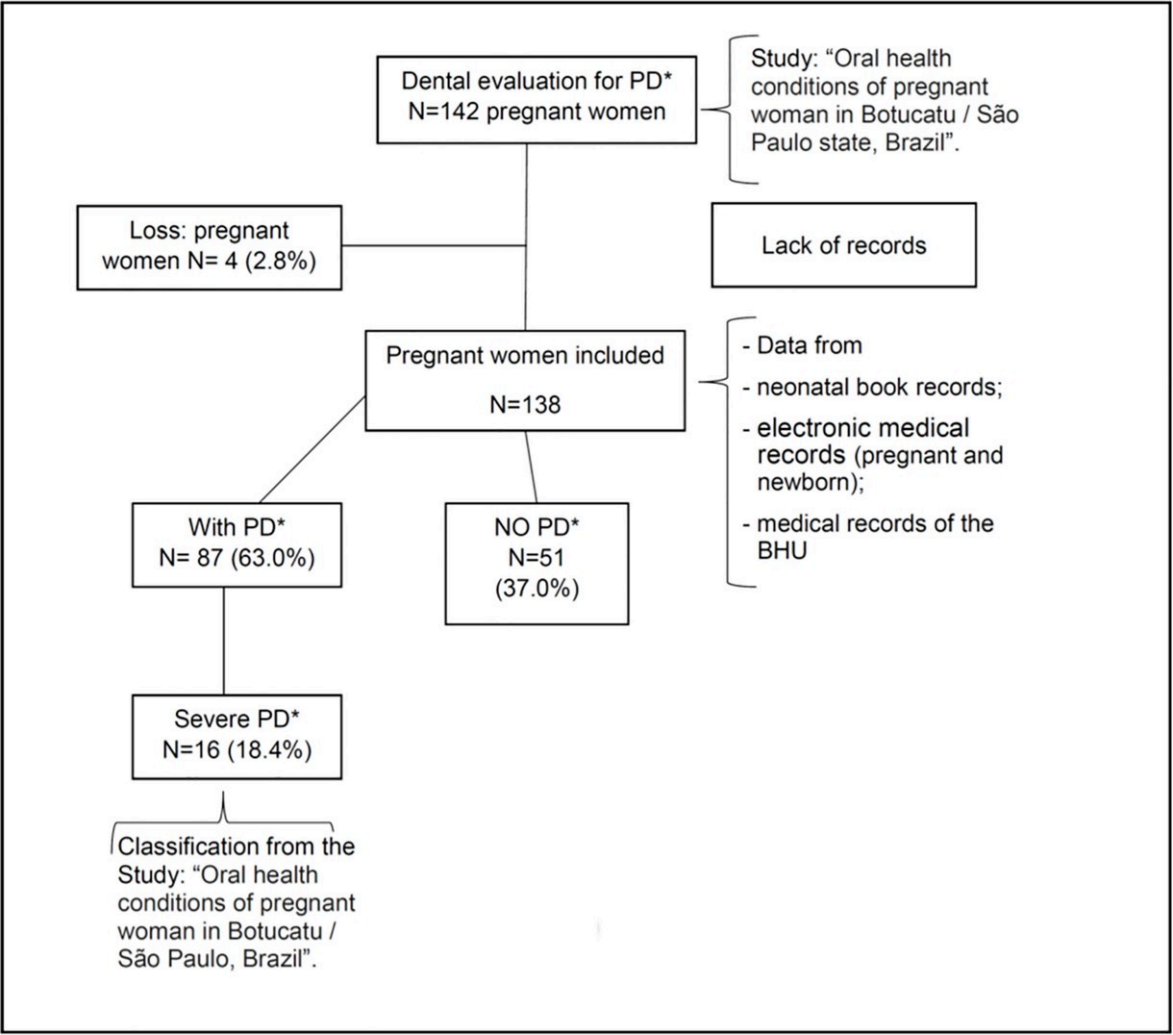
Flowchart of data collection and inclusion of pregnant women. * PD = periodontal disease.

Considering the sample of 142 pregnant women, the variables selected for the study were maternal (age, years of study), gestational (time, type of pregnancy, diseases, intercurrences), delivery (cesarean or normal) and newborn (weight, sex, gestational age, Apgar score, malformation, intercurrences and neonatal hospitalization, death or not). All information was collected from medical records of the BHU, Botucatu Clinics Hospital and the Information System for Live Births (SINASC) of the Ministry of Health (Fig 1).

At the end of the collection of these data, the classification of the groups of interest was made. Considering the exposure or not to Periodontal Disease, the newborns were stratified into two groups: offspring of mothers with PD (subdivided for Severe Periodontal Disease—SPD) and offspring of mothers without PD.

### Standard protocol approvals and patient consents

This study received approval from the Botucatu Medical School Research Ethics Committee (48596815.6.0000.5411 / 2015). Written informed consent was obtained from all patients participating in the study.

### Clinical definitions

- Community Periodontal Index (CPI): A smooth blunt periodontal probe from the WHO (World Health Organization) was used. The presence of calculi and periodontal pockets on the buccal and lingual surfaces of six teeth were identified;
- Small, Adequate or Large classification of the gestational age (SAG, AGA and LGA) according to Fenton intrauterine growth curve [9];
- Low Birth Weight: <2500g, Very Low Birth Weight <1500g and Extreme low birth weight: <1000g;
- Fetal Growth Restriction from Beune *et al*. was defined for the infants who presented: Birth weight lower than Percentile 3 or three of the following changes: birth weight <P10; Cephalic perimeter <P10; Length <P10; prenatal diagnosis of intrauterine growth restriction and maternal comorbidities known to impact the fetal growth [10].

### Statistical analysis

The Chi-square test or Fisher exact test was used for the comparison between the study groups, taking into consideration socio-demographic characteristics, gestational and delivery conditions, as well as birth and newborn conditions [11].

Adjusted multiple logistic regression models were also applied to analyse the chance of occurrence of maternal and newborn negative outcomes, as a result of periodontal disease, correcting the effects of confounding variables. Associations were considered statistically significant for p-value <0.05. Analyzes were carried out in SPSS v21.0 software.

The potential confounders pointed out in the literature for maternal outcomes were: maternal age, maternal level of education, number of previous pregnancies and smoking. For the premature rupture of the membrane outcome, the urinary tract infection and vulvovaginitis were also included as potential confounders.

For neonatal outcomes, the confounders were: maternal age; maternal level of education; number of previous pregnancies; smoking; hypertensive disease; gestational diabetes; pre-eclampsia and eclampsia; urinary tract infection; vulvovaginitis; third trimester hemorrhage and premature rupture of the membrane.

## Results

### Demographic characterization

Among the 142 pregnant women and their respective newborns, only 4 pregnant women did not have a medical record available, restraining the data loss of the study to 2% of its total. These pregnant women probably joined particular healthcare plans or moved out from Botucatu/SP and, therefore, were excluded from the study.

**Fig 2:**
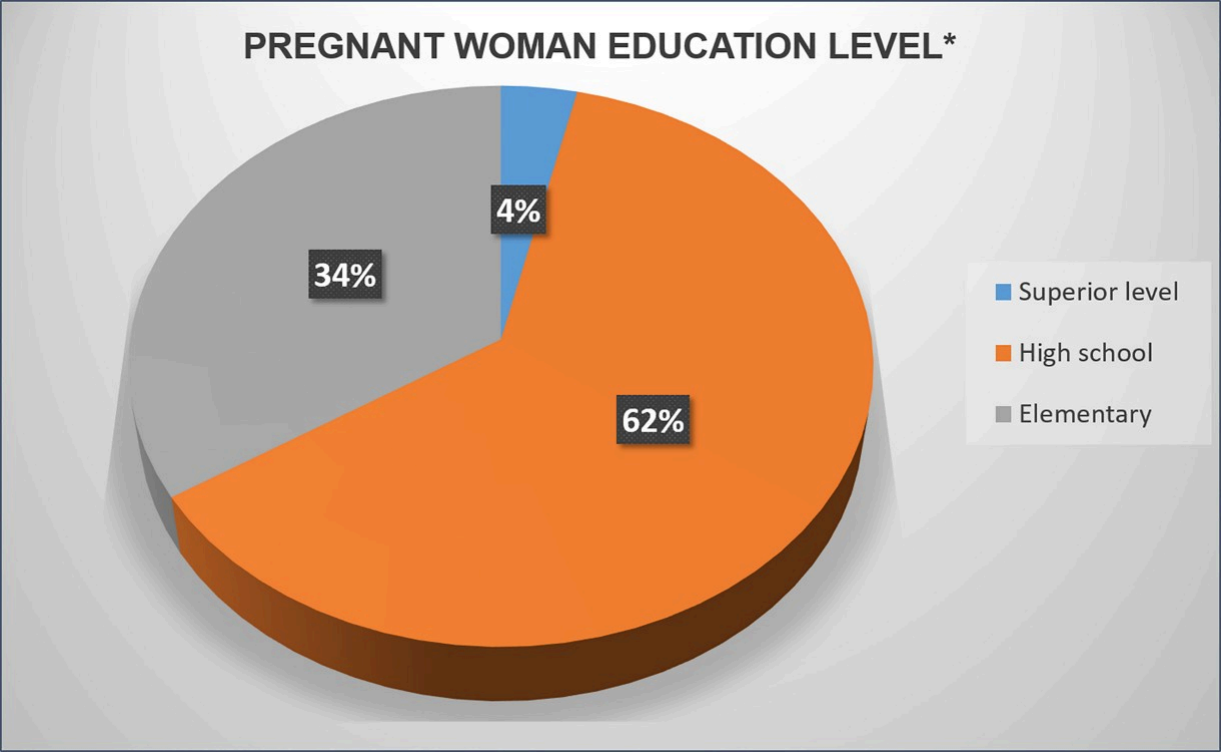
Distribution of level of education of pregnant women in the study.

All the pregnant women had a single fetus gestation and the average maternal age was 25 years (PD = 6.59), ranging from 14 to 44 years. The level of education is indicated in the Fig 2, showing a small percentage of pregnant women with superior level of education (3.6%) and a majority (62.3%) with high school level.

### Diagnosis of periodontal disease

The frequency of pregnant women with periodontal disease during gestation was 63.00% (n = 87), of which 18% (n = 16) with PD in their severe form (SPD). Gestational age at the time of PD diagnosis ranged from 16 to 39 weeks, with an average of 26.5 weeks (PD = 6.51). The percentage of evaluations and diagnoses of PD performed in the second and third trimester of gestation were 53.6% and 46.4%, respectively.

### Gestational and labor conditions

The cesarean index was approximately 50% in pregnant women with or without PD (p = 0.83) or SPD (p = 0.94), with no statistical difference between these study groups.

The relation between exposure and absence of periodontal disease during gestation and gestational intercurrences are shown in Table 2. It was observed that there was no statistically significant differences between the studied factors and their association with different study groups.

**Table 2:** Maternal, gestational and delivery variables, according to study groups (n = 138), Botucatu / SP, 2012–2014.

Variable	No PD* (n = 51)	PD* (n = 71)	Severe PD (n = 16)	*P*
	N (%)	N (%)	N (%)	
Age^1^				
<20 years	10 (19.6)	13 (18.3)	3 (18,8)	*0*.*992*
20–34 years	36 (70.6)	49 (69.0)	11(68.7)	
> = 35 years	5 (9.8)	9 (12.7)	2 (12.5)	
Schooling^2^				
≤12 years	18 (35.3)	21 (29.6)	8 (50.0)	*0*.*289*
>12 years	33 (64.7)	50 (70.4)	8 (50.0)	
N.Pregnancy ^2^.				
Only one	24 (47.1)	33 (4.5)	5 (31.2)	*0*.*708*
≥ 2	27 (52.9)	38 (53.5)	11 (6.8)	
Cesarean	19 (39.2)	28 (3.4)	6 (37.5)	*0*.*968*
Smoking	5 (9.8)	11 (15.5)	4 (25.0)	*0*.*303*
Hypertension Gestational	3 (5.9)	4 (5.6)	1 (6.2)	*0*,*095*
Diabetes	1 (1.9)	1 (1.4)	0 (0.0)	*0*.*848*
Urinary Infecction	11 (21.6)	20 (28.2)	7 (43.7)	*0*.*200*
Vulvovaginitis	8 (15.7)	12 (16.9)	6 (37.5)	*0*.*126*
3^th^ trimester bleeding	3 (5.9)	8 (11.3)	2 (12.5)	*0*.*546*
Pre eclampsia	2 (3.9)	2 (2.8)	2 (12.5)	*0*.*225*
Eclampsia	1 (1.9)	1 (1.4)	1 (6.2)	*0*.*483*
PRM^4^	7 (13.7)	21 (29.6)	6 (37.5)	*0*.*060*

* PD = Periodontal disease

** SPD = severe PD

^1^ according to age groups 1, 2 or 3

^2^ according to the classification defined in the method

^3^ UTI = Urinary tract infection

^4^ PRM = premature rupture of the membrane.

Table 3 shows the relation between exposure to PD and maternal outcomes during pregnancy and at the time of delivery, considering the potential confounders. It was possible to identify a 3.45 times higher chance of vulvovaginitis (p = 0.050) and 5.59 times higher chance of premature rupture of the membrane (p = 0.017) in pregnant women exposed to SPD, in comparison with those without exposure to periodontal disease.

**Table 3:** Maternal outcomes according to maternal exposure to periodontal disease (PD) and severe periodontal disease (SPD), Botucatu / SP, 2012–2104 (n = 138).

Outcome	Periodontal disease (PD*)		Severe PD**	
	OR^1^ (IC 95%)	*P*	OR^1^ (IC 95%)	*P*
UTI^2^^,^^3^	1.34 (0.57–3.18)	*0*.*496*	2.44 (0.72–8.30)	*0*.*150*
Vulvovaginitis^3^	1.06 (0.39–2.88)	*0*.*901*	3.45 (0.93–12.79)	***0*.*050***
3^th^ trimester bleeding ^3^	2.07 (0.51–8.45)	*0*.*307*	1.89 (0.26–13.42)	*0*.*523*
Premature rupture of membrane^,^^4^	2.62 (0.96–7.11)	*0*.*058*	5.59 (1.36–22.92)	***0*.*017***

* PD = Periodontal Disease

** SPD = severe PD

^1^ Reference category was the absence of PD

^2^ UTI = Urinary tract infection

^3^ corrected for maternal age (years), level of education, number of pregnancies and smoking

^4^ also corrected for UTI and vulvovaginitis.

### Intercurrences at birth and consequences for the newborn

The relation between exposure to periodontal disease and severe PD, and the conditions of birth and neonatal evolution are shown in Table 4. It was possible to observe that there was no statistically significant difference between the study groups.

**Table 4:** Presence of the variables at birth and during the neonatal period, according to study groups (n = 138), Botucatu / SP, 2012–2014.

Variable	No PD* (n = 51)	PD* (n = 71)	Severe PD** (n = 16)	*P*
	N (%)	N (%)	N (%)	
Low birth weight	2 (3.9)	7 (9.8)	3 (18.7)	*0*.*163*
Prematurity	2 (3.9)	5 (7.0)	3 (18.7)	*0*.*136*
Fetal growth restriction	1 (1.9)	6 (8.4)	3 (18.7)	*0*.*066*
Small for GA^1^	9 (17.6)	16 (22.5)	4 (25.0)	*0*.*741*
Apgar 1^st^ min (<7)	5 (9.8)	9 (12.7)	2 (12.5)	*0*.*881*
Neonatal hospitalization	4 (7.8)	7 (9.8)	2 (12.5)	*0*.*843*
Neonatal infeccion	1 (1.9)	2 (2.8)	0.0	*0*.*777*
Neonatal death	2 (3.9)	1 (1.4)	0.0	*0*.*526*

* PD = Periodontal Disease

** SPD = severe PD

^1^ GA = Gestational Age.

Table 5 shows the relation between exposure to PD in the groups and neonatal outcomes considering their potential confounders. The chance of fetal growth restriction was 11 times higher (p = 0.041) in pregnant women exposed to SPD—in comparison to those without exposure to periodontal disease.

**Table 5:** Neonatal outcomes according to maternal exposure to periodontal disease (PD) and severe periodontal disease (SPD), Botucatu / SP, 2012–2104 (n = 138).

Outcome	Periodontal disease (PD)		Severe PD	
	OR^1^ (IC 95%)	*P*	OR^1^ (IC 95%)	*P*
Prematurity^2^	1.65 (0.29–9.33)	*0*.*571*	5.62 (0.71–38.50)	*0*.*102*
Low birth weight^2^	2.93 (0.46–2.36)	*0*.*298*	4.81(0.68–33.92)	*0*.*115*
Fetal growth restriction^2^	4.61 (0.53–39.57)	*0*.*163*	11.53 (1.10–12.26)	***0*.*041***
Small for GA*^2^	1.39 (0.52–3.69)	*0*.*503*	1.32(0.29–5.91)	*0*.*711*

^1^ Reference category was the absence of PD

^2^ corrected for maternal age (years), level of education, number of gestations, smoking, gestational diseases, hemorrhage and premature rupture of membrane

* GA = gestational age.

## Discussion

The periodontal disease in pregnancy starts with a dental plaque and is accentuated by the action of hormones, mainly estrogen and progesterone, increased during pregnancy. These hormones trigger greater vulnerability of dental tissues to PD, mainly due to edema and increased vascularity of dental tissue [12]. Therefore, pregnant women with PD may develop the most severe form of the disease until the end of gestation [13]. This study evidenced a high prevalence of periodontal disease among pregnant women, and the rates of the severe form of PD are also of great concern.

More over, here is a relation between chronic diseases with low inflammation and the increased risk of developing gestational and neonatal comorbidities, which can be aggravated when they are associated with PD [5,14].

It is known today that periodontal disease is initiated and perpetuated by a small group of bacteria, predominantly gram-negative, anaerobic or microaerophilic, that colonize the subgingival area; being the most common human periodontitis caused by Porphyromonas gingivalis, Bacteroides forsythus and Actinobacillus actinomycetemcomitans [15]. These infections can generate lipopolysaccharide reservoirs, triggering the release of interleukin 1 beta and prostaglandin E2, targeting the placental membranes through the bloodstream; therefore, periodontal infection in pregnant women becomes a risk factor for premature low birth weight [3].

Thus, we consider this pathophysiology responsible for the increase in the risk of pregnant woman with SPD for the unfavorable neonatal outcomes (such as fetal growth restriction, which is mainly associated with high fetal and neonatal morbimortality, neurological sequelae and unsatisfactory development); implying high costs for the healthcare assistance, since a large percentage of these newborns require more advanced treatments, and human resources [16].

Unlike the results of our study, the literature evidences the association of PD with preterm birth [17–19]. Offembacher *et al*. found significantly higher levels of prostaglandin E in the gingival fluid of mothers of preterm low birth weight infants. In addition, they also found a significant inverse association between birth weight and prostaglandin-E levels [15]. This could be an explanation for the association between periodontal disease and fetal growth restriction.

From the relation between invasive bacteria and hostage, there may be some organic repercussions [16]. Chronic exposures to inflammatory mediators may directly affect the development of the fetus and suggest the possibility of a causal relation between maternal periodontal disease and low birth weight. Consonant with our study, Ananth *et al*. showed the association between PD and fetal growth restriction—being the association more relevant in the presence of the severe form of PD [20].

Soucy-Giguère and Gonzalez-Jaranay *et al*. state a greater probability that pregnant women with PD can develop pre-eclampsia [21,13]. Chanomethaporn on the other hand, suggests the association between PD and spontaneous abortion. These associations, however, were not found in this study [22].

As in the work of Sobel, we found a high prevalence of vulvovaginitis during gestation with increased odds for this event in pregnant women with SPD [23]. This diagnosis is very important because there is an association of vulvovaginitis, even with adequate treatment, and a significant increase in the risk of miscarriage and premature birth [24].

Even though premature rupture of the membrane (found in our study with a higher chance of occurring in pregnant women with PD and SPD) is often related to vulvovaginitis and also urinary tract infection during pregnancy [24], it was possible to dismiss these two infections as potential confounders in the statistical analysis, and affirm they did not behave as factors associated with the increased chances of premature rupture of the membrane; which may actually be associated with PD and SPD in the pregnant women studied.

The maternal level of education above 12 years can be considered a good level of education among pregnant women attending Botucatu's Basic Health Units. Some studies, such as Nascimento, Haiddar *et al*. and Baldani found greater use of dental services by patients with higher educational level. In our study we can confirm the PD diagnosis was given after a dental evaluation and state that the consultation occurred mostly in the second trimester of pregnancy [25–27]; however we could not retrieve the indicated treatment and the follow-up of these pregnant women in the dental services available in the primary health care of Botucatu. According to Al Habashneh, few pregnant women usually use the dental health service available, 49% of parturients who had dental follow-up in their study were aware of the association between periodontal disease and negative repercussions in gestation [28]. In addition, Sousa states that the majority of pregnant women (98%) have been poorly oriented in how to promote oral health and this percentage was significantly associated with the presence of periodontal disease [29].

It is important to emphasize that not only the pregnant women are unaware of the relation between periodontal disease and gestational and neonatal repercussions, but almost every health professionals; which lowers the chances of diagnostic and prevention of maternal, gestational and newborn complications [30,31]. Moreover, Gupta highlights the necessity of better training the health professionals, specially doctors, about oral health of the pregnant woman [32].

The Brazilian Ministry of Health emphasizes the importance of hygiene and oral care for pregnant women, suggesting a healthier diet and frequent brushing, with at least one dentist appointment during pregnancy (to registered in the Pregnant Woman Portfolio). In the material available for all brazilian health care system, the Ministry of Health recommends guidelines and dental care procedures during pregnancy to help protect the health of the embryo, the fetus and the pregnant woman [33,34].

We consider important the possible relation between the difficulty of self-care attention during the gestation and the development of periodontal disease, as well as the vaginosis.

Shimizuet *et al*. observed that in Brazil, the social representation of the pregnant women on the gestational process is still displayed as a natural phenomenon, contributing to the lack of care during pregnancy, the non-adherence and even avoidance of prenatal programs, and the high incidence of severe gestational disorders [35]. These results indicate the necessity to review and expand the access of pregnant women to health services, as well as the urge to improve the quality of consultations—especially by strengthening the reception of the pregnant women and ensuring the adherence to the prenatal programs.

A very important topic already under discussion in our country for the past several years is the high rate of cesarean deliveries among pregnant women [36], regardless of the exposure to periodontal disease—remembering that most of the prenatal follow-up in the primary health care are rated as habitual risk and can be confirmed as such with the verification of the low incidence of hypertensive complications and diabetes. According to the WHO, the ideal rate of cesarean section would be between 10% and 15%. When performed for medical reasons, caesarean sections can reduce maternal and perinatal mortality and morbidity, but there is no evidence that its unnecessary indication brings benefits. Moreover, in many cases, it exposes the parturient to immediate risks, such as infections, and, in the long term, negatively affecting future pregnancies [36].

The reduced size of the sample size was one constraint of the research and there may be other associations not identified between PD and maternal or neonatal events—which could be possibly seen with a larger number of participants. Another limitation that should be considered is the low prevalence of some unfavorable maternal outcomes found in the literature as potential repercussions of PD, which in our study had to be discarded for statistical analysis. We also considered that the uncertainty regarding the treatment of periodontal disease in pregnant woman and the diagnosis in the third trimester of gestation as a limitation to interpret the results found.

There are still few studies about the association of periodontal disease in gestation and its repercussions due to the difficulty to achieve an adequate diagnosis during the gestational period, as well as the complications involved in the evaluation of the inflammatory mediators that could prove the pathophysiological association between them. However, the identification of this health problem, which is clearly avoidable and treatable, during prenatal follow-up is an important milestone, and thus demonstrates the relevance of this study, which found unfavorable outcomes for the mother-infant dyad.

The implication of increased odds for premature rupture of the membrane and fetal growth restriction could not be minimized, although more studies could confirm and better explain these associations.

Based on the results of this study, it is our intention to stimulate knowledge, practices and the perception that the oral health of the pregnant women is very relevant to avoid negative repercussions for the parturient and the newborn. We also intend to promote enhancements in the prevention and treatment of periodontal disease during prenatal follow-up, considering its impact on maternal and child health care.

## Conclusions

Severe Periodontal Disease increased the chance of neonatal and maternal negative outcomes, such as fetal growth restriction, vulvovaginitis and premature rupture of the membrane “S1 and S2 Tables”.

Our results highlight the importance of these studies on periodontal diseases during pregnancy and the relevance of evaluating oral health during prenatal care.

We believe that oral health should be an important focus in the follow-up of pregnant women in every maternal and child health services, thus incorporating the diagnosis of maternal oral health and Periodontal Disease as a possible risk factor for the health of the mother and the newborn.

## Supporting information

S1 Table Maternal outcomes according to maternal exposure to periodontal disease (PD) and severe periodontal disease (SPD), Botucatu / SP, 2012–2104 (n = 138). (PDF)

S2 Table Neonatal outcomes according to maternal exposure to periodontal disease (PD) and severe periodontal disease (SPD), Botucatu / SP, 2012–2104 (n = 138). (PDF)

S1 Dataset (XLSX)
