# Supplementary material for: Periodontal disease: Repercussions in pregnant woman and newborn health—A cohort study
Source: PLoS One. 2019 Nov 22;14(11):e0225036. doi: 10.1371/journal.pone.0225036 (PMC6874354; doi:10.1371/journal.pone.0225036)
Supplement: S1 Table — (PDF) [file pone.0225036.s001.pdf]

Table 3 - Maternal outcomes according to exposure to periodontal disease (PD) and severe periodontal disease (SPD), Botucatu city, São Paulo state, Brazil, 2016-2017 (n=138).

	**Periodontal disease (PD)**		**Severe PD**	
**Outcome**	**OR[1] (IC 95%)**	***P***	**OR[1] (IC 95%)**	***P***
UTI[2;3]	1.34 (0.57-3.18)	*0.496*	2.44 (0.72-8.30)	*0.150*
Vulvovaginitis[3]	1.06 (0.39-2.88)	*0.901*	3.45 (0.93-12.79)	*0.063*
3th trimester bleeding [3]	2.07 (0.51-8.45)	*0.307*	1.89 (0.26-13.42)	*0.523*
Premature rupture of membrane[,4]	2.62 (0.96-7.11)	*0.058*	5.59 (1.36-22.92)	***0.017***

* PD = Periodontal Disease; ** SPD = severe PD;

[1] Reference category in the regression analysis was the absence of PD; [2] UTI = Urinary tract infection;[3] corrected for maternal age (years), level of education, number of pregnancies and smoking; [4] also corrected for UTI and vulvovaginitis.
