# Supplementary material for: Periodontal disease: Repercussions in pregnant woman and newborn health—A cohort study
Source: PLoS One. 2019 Nov 22;14(11):e0225036. doi: 10.1371/journal.pone.0225036 (PMC6874354; doi:10.1371/journal.pone.0225036)
Supplement: S2 Table — (PDF) [file pone.0225036.s002.pdf]

Table 5 - Neonatal outcomes according to maternal exposure to periodontal disease (PD) and severe periodontal disease (SPD), Botucatu city, São Paulo state, Brazil, 2016-2017 (n = 138).

	Periodontal disease (PD)		Severe PD	
**Outcome**	**OR[1] (IC 95%)**	***P***	**OR[1] (IC 95%)**	***P***
Prematurity[2]	1.65 (0.29-9.33)	*0.571*	5.62 (0.71-38.50)	*0.102*
Low birth weight[2]	2.93 (0.46-2.36)	*0.298*	4.81(0.68-33.92)	*0.115*
Fetal growth restriction[2]	4.61 (0.53-39.57)	*0.163*	11.53 (1.10-12.26)	***0.041***
Small for GA*[2]	1.39 (0.52-3.69)	*0.503*	1.32(0.29-5.91)	*0.711*

[1] Reference category in the regression analysis was the absence of PD;

[2] corrected for maternal age (years), level of education, number of gestations, smoking, gestational diseases, hemorrhage and premature rupture of membrane; * GA = gestational age.
